# Effects of ranitidine and nizatidine on the risk of gastrointestinal cancer

**DOI:** 10.3389/fonc.2023.1182174

**Published:** 2023-07-27

**Authors:** Hyejung Kang, Chung Mo Nam, Dong-Woo Choi, Sohee Park

**Affiliations:** ^1^ Department of Health Informatics and Biostatistics, Graduate School of Public Health, Yonsei University, Seoul, Republic of Korea; ^2^ Department of Preventive Medicine, Yonsei University College of Medicine, Seoul, Republic of Korea; ^3^ Cancer Big Data Center, National Cancer Control Institute, National Cancer Center, Goyang, Republic of Korea

**Keywords:** gastrointestinal cancer, H2-receptor antagonists, ranitidine, nizatidine, National Health Insurance Service-National Sample Cohort, nationwide health claims data, retrospective cohort study, nested case-control study

## Abstract

**Purpose:**

Gastrointestinal (GI) cancer occurs in digestive organs such as the stomach, colon, liver, esophagus, and pancreas. About 83,034 cases occurred in Korea alone in 2020. Dietary factors, alcohol consumption, *Helicobacter pylori* (*H. pylori*), and lifestyle factors increase the incidence of diseases such as gastritis, peptic ulcer, pancreatitis, and gastroesophageal reflux disease (GERD), which can develop into GI cancer. However, in 2019, the US Food and Drug Administration announced that the drugs ranitidine and nizatidine, which are used for digestive disorders, contain carcinogens. In this study, we investigated the effects of ranitidine and nizatidine on the development of GI cancer.

**Materials and methods:**

In this study, using National Health Insurance Service–National Sample Cohort (NHIS-NSC) version 2.5 (updated from 2002 to 2019), subjects who developed GI cancer were enrolled in the case group, and those who were at risk of, but did not develop, cancer were enrolled in the control group. Thereafter, risk-set matching was performed (1:3 ratio) by sex and age at the time of diagnosis of cancer in the case group. Through this procedure, 22,931 cases and 68,793 controls were identified. The associations of ranitidine and/or nizatidine with GI cancer were confirmed by adjusted odds ratios (aORs) and 95% confidence intervals (CIs) calculated through conditional logistic regression analysis.

**Results:**

The aORs of ranitidine and/or nizatidine users were lower than those of nonusers in all average prescription days groups (< 30 days/year: aOR [95% CI] = 0.79 [0.75-0.82]; 30–59 days/year: aOR [95% CI] = 0.66 [0.59-0.73]; 60–89 days/year: aOR [95% CI] = 0.69 [0.59-0.81]; ≥ 90 days/year: aOR [95% CI] = 0.69 [0.59-0.79]). Sensitivity analyses were conducted with different lag periods for the onset of GI cancer after drug administration, and these analyses yielded consistent results. Additional analyses were also performed by dividing subjects into groups based on cancer types and CCI scores, and these analyses produced the same results.

**Conclusion:**

Our study, using nationwide retrospective cohort data, did not find evidence suggesting that ranitidine and nizatidine increase the risk of GI cancer. In fact, we observed that the incidence of GI cancer was lower in individuals who used the drugs compared to nonusers. These findings suggest a potential beneficial effect of these drugs on cancer risk, likely attributed to their ability to improve digestive function.

## 1 Introduction

The risk of gastrointestinal (GI) cancers is high in most Asian countries. According to a previous study, GI cancers were commonly found to develop in the colon, rectum, stomach, liver, esophagus, and pancreas (1). The incidence of GI cancer was approximately 3.2 million in Asia and 0.08 million in Korea in 2020 (2). Diet, alcohol consumption, smoking, and other lifestyle factors increase the risk of GI cancers (3–7).

A meta-analysis conducted in Korea and Japan showed that habitual consumption of salted vegetables increases the risk of stomach cancer (3). In addition, the incidence of colorectal cancer is increasing in association with the adoption of Western eating habits (4). Liver cancer, like other digestive cancers, is prevalent in East Asia. Although lifestyle factors such as smoking and drinking alcohol can promote liver cancer, hepatitis B virus (HBV) and hepatitis C virus (HCV) infections are the most well-known causes (5). Men are at higher risk of esophageal cancer than women. Moreover, smoking and alcohol consumption, and reduced intake of fresh vegetables, increase the risk of developing gastroesophageal reflux disease (GERD) (6). A clear cause for pancreatic cancer has not yet been identified, although smoking, alcohol consumption, eating habits, body mass index (BMI), sex, age, and diabetes mellitus (DM) may all play a role. It is very important to elucidate the epidemiological mechanisms of cancers and identify additional risk factors (7).

In September 2019, the US Food and Drug Administration (FDA) announced the detection of a carcinogen called N-nitrosodimethylamine (NDMA) in two drugs used to treat GI diseases: ranitidine and nizatidine. These drugs belong to the H2-receptor antagonist (H2RA) family, and have primarily been used to treat gastric ulcers and GERD. Ranitidine generally absorbed in the small intestine, and the duration of action lasts about 8 to 12 hours (8). It has a half-life of 2.5 hours (9). Nizatidine, which is a hybrid structure of ranitidine and famotidine, is also absorbed into the body in a manner similar to that of ranitidine, and its effect lasts about 8 hours (10). The elimination half-life is between 1.1 and 1.6 hours (11). The availability of these drugs as either over-the-counter (OTC) or prescription drugs depends on the dosage. The safe level of NDMA specified by the FDA is 0.32 parts per million (ppm), but according to laboratory data, most ranitidine and nizatidine samples had higher levels (12). Thus, the drugs were withdrawn from the market. NDMA is mainly used for industrial purposes, and is harmful to the human body, causing severe irritation to the eyes, vomiting, or abnormal liver function when ingested through inhalation, intake, or skin contact (13). The intake of this component was found to increase the risk of liver cancer, kidney cancer, and lung cancer as well as GI cancer. It also showed the same results in both rodents and humans (14, 15).

However, in subsequent studies, it could not be conclusively proven that ranitidine and nizatidine increased the risk of cancer. One of the previous studies using health claims data showed no association between gastric cancer incidence and ranitidine and nizatidine intake. As a result of the Cox proportional hazards regression analysis, no statistically significant results were obtained in the case group (hazard ratio, HR [95% confidence interval, CI] = 1.02 [0.87-1.20]) and the other H2RAs intake group (HR [95% CI] = 1.00 [0.85-1.17]) compared to the comparison group (16). Also, the study analyzing the risk of different cancers (liver, colorectal, biliary, stomach, lung, prostate, kidney, bladder, uterine, breast, and thyroid) did not produce significant results (overall: HR [95% CI] = 0.99 [0.91-1.07]) (17). Therefore, in this study, we used retrospective cohort data from the Republic of Korea to investigate the effects of ranitidine and nizatidine intake on the likelihood of development of GI cancer in the Korean population using nested case-control study design.

## 2 Materials and methods

### 2.1 Dataset and study design

National Health Insurance Service (NHIS) is a nationwide service that includes the majority of the Korean population. We analyzed the National Health Insurance Service-National Sample Cohort (NHIS-NSC; version 2.5) which represents 2.2% of the total Korean population. The NHIS stratified the cohort based on various factors such as sex, age, social security status, income level, and residential location, ensuring a representative sampling (18). The NHIS-NSC data were collected from 2002 to 2019.

A nested case-control study design was used to investigate the association of exposure to ranitidine and nizatidine with GI cancer. This design was used to reduce selection and immortal time biases, and because it is well suited to pharmacoepidemiologic studies that follow up many subjects over time and investigate time-dependent exposure (19).

### 2.2 Study population

**Figure 1:**
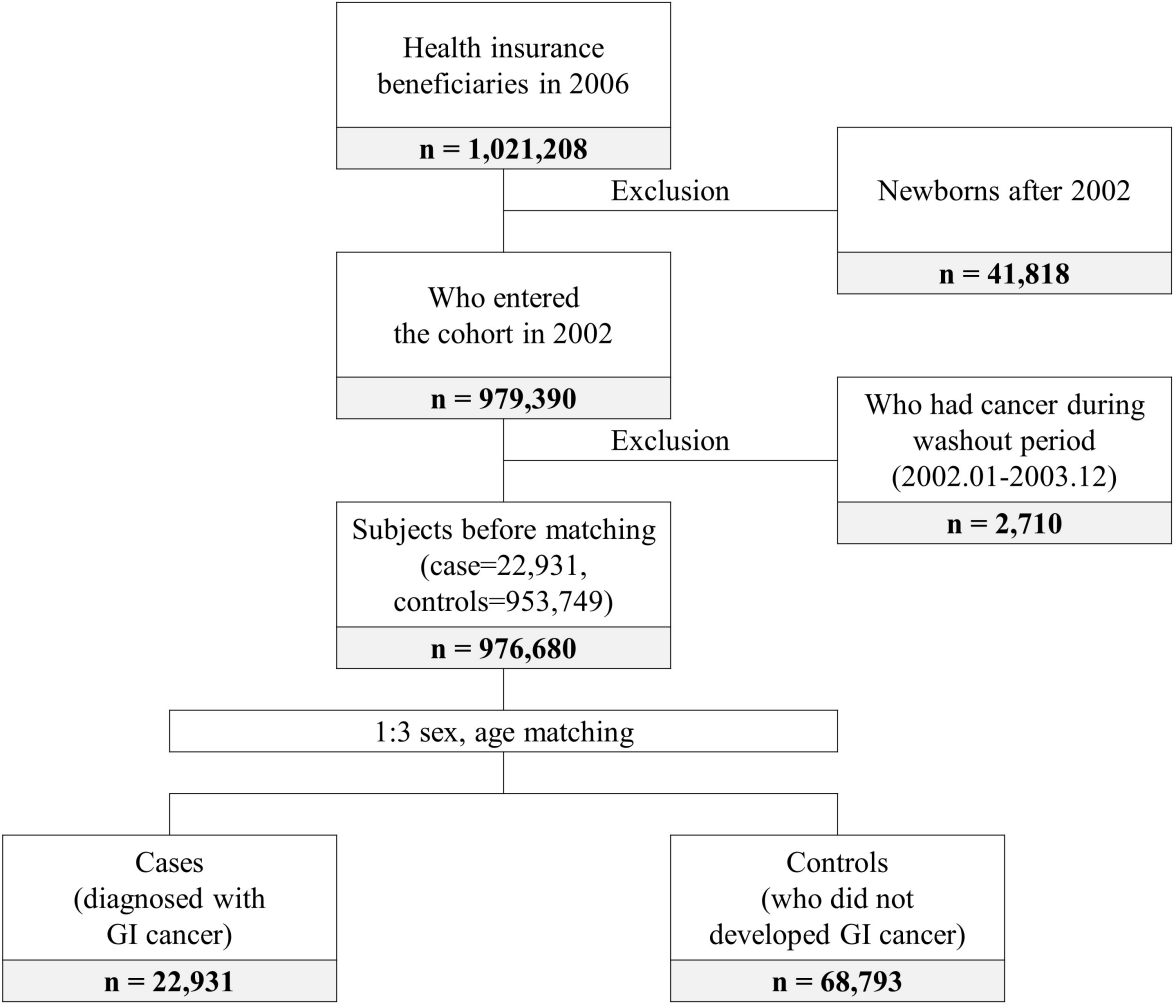
Flow chart of study population selection.

The patients in this study all developed GI cancer between 2004 and 2019. The controls were individuals who did not develop cancer during that period. Cases and controls were matched in the year when GI cancer occurred. In total, 979,390 subjects entered the cohort in 2002. Subjects who had cancer during the 2-year washout period were excluded. Then age and sex matching (1:3 ratio) were performed. Finally, 22,931 cases and 68,793 controls were included in this study (**Figure 1**).

### 2.3 Outcome and variable definitions

Only cases of GI cancer that occurred during the follow-up period were included in our outcome analysis. International Classification of Diseases, 10^th^ revision (ICD-10) codes were used to determine cancer incidence (colorectal cancer: C18, C19, and C20; stomach cancer: C16; liver cancer: C22; esophageal cancer: C15; pancreatic cancer: C25).

The main variable of interest was the average prescription days per year, i.e., the total number of prescription days of ranitidine and nizatidine divided by the time between the start date of the cohort and diagnosis of GI cancer multiplied by 365 days (20). A lag period of 180 days (6 months) was considered for that period (21); to test the accuracy of the lag period, sensitivity analysis was performed, which also included 365- and 730-day lag periods. Drug classification codes provided by the Health Insurance Service Review & Assessment (HIRA) service were used to identify the drugs.

Sociodemographic status (residential location, social security status, income level, disability); the presence of hypertension, DM, and dyslipidemia at the time of GI cancer; the Charlson Comorbidity Index (CCI) during the year before cancer diagnosis (based on a variety of diseases including myocardial infarction, congestive heart failure, peripheral vascular disease, cerebrovascular disease, dementia, chronic pulmonary disease, rheumatologic disease, peptic ulcer disease, mild liver disease, diabetes without chronic complication, diabetes with chronic complication, hemiplegia or paraplegia, renal disease, moderate or severe liver disease, and AIDS/HIV); and the average number of prescription days per year for proton pump inhibitors (PPIs) and nonsteroidal anti-inflammatory drugs (NSAIDs) during the same period as that considered for ranitidine and nizatidine were used as covariates in the analyses.

### 2.4 Statistical analysis

Descriptive statistics of the subjects’ characteristics were generated using a chi-squared test. Univariable and multivariable conditional logistic regression analyses were performed to investigate the effects of ranitidine and nizatidine on the development of GI cancer. Sensitivity analyses (by lag period) and additional analyses (by five cancer types and CCI scores) were also performed. All statistical tests were two-sided, and the significance level was set at *p <* 0.05. SAS Enterprise Guide software (version 7.1; SAS Institute, Cary, NC, USA) was used for the analyses.

## 3 Results

### 3.1 Subjects’ characteristics

**Figure 2:**
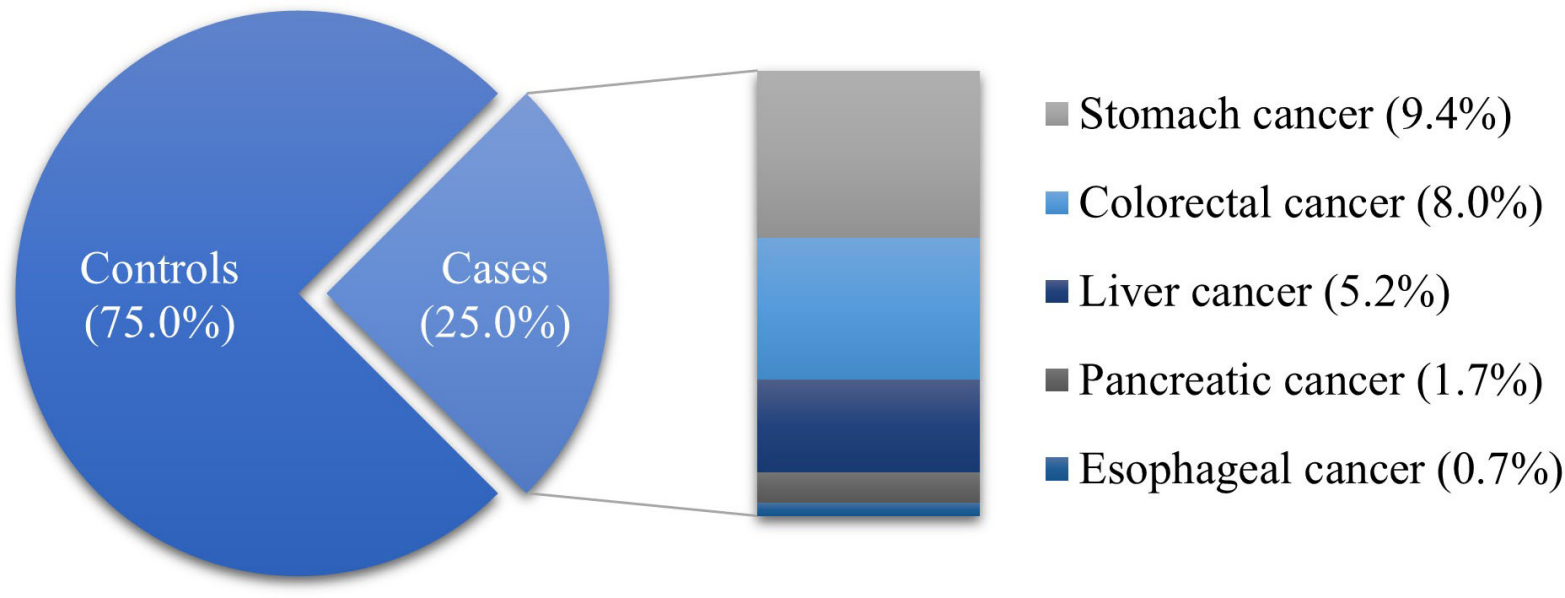
Distribution of GI cancers (*N* = 91,724).

The proportion of ranitidine and/or nizatidine users was higher than that of nonusers in both the case (61.5%) and control (60.6%) groups. Among the drug users, the proportion who took drugs for < 30 days was the highest. The proportion of subjects living in rural areas was higher than those living in urban areas (cases, 80.0%; controls, 78.9%). A large proportion of subjects with corporate insurance in both groups (cases, 61.2%; controls, 61.5%). Also, the proportion of subjects with high income was higher than other income levels in both groups (cases, 31.3%; controls, 32.4%). Most of the cases had a CCI ≥ 3 (59.0%), while most of the controls had a CCI of 0 (43.0%). There were fewer PPI users than nonusers in both groups (cases, 32.9%; controls, 33.9%). The majority of subjects had experience taking NSAIDs (cases, 96.2%; controls, 95.6%). The proportion of subjects with disability (cases, 12.8%; controls, 12.2%), hypertension (cases, 29.3%; controls, 33.1%), DM (cases, 19.1%; controls, 14.2%) or dyslipidemia (cases, 10.0%; controls, 12.4%) were lower than who were not. The distribution of case and control groups was significantly different in all variables except sex, and age (**Table 1**). Stomach cancer (37.5%) was the most common type of GI cancer in the case group, followed by colorectal (31.9%), liver (20.7%), pancreatic (6.9%), and esophageal cancers (2.7%) (**Figure 2**).

**Table 1:** Sociodemographic characteristics of the subjects.

Variables, *N* (%)	Cases		Controls		*p* ^a^
	(*N* = 22,931)		(*N* = 68,793)		
Ranitidine and/or Nizatidine					0.007
Nonusers	8,838	(38.5)	27,078	(39.4)	
<30 days/year	12,560	(54.8)	37,479	(54.5)	
30-59 days/year	786	(3.4)	2,275	(3.3)	
60-89 days/year	352	(1.5)	898	(1.3)	
≥90 days/year	395	(1.7)	1,063	(1.6)	
Sex					1.000
Male	14,896	(65.0)	44,688	(65.0)	
Female	8,035	(35.0)	24,105	(35.0)	
Age					0.942
<20 years old	9	(0.0)	30	(0.0)	
20-29 years old	65	(0.3)	201	(0.3)	
30-39 years old	583	(2.5)	1,843	(2.7)	
40-49 years old	2,274	(9.9)	6,898	(10.0)	
50-59 years old	4,969	(21.7)	14,847	(21.6)	
60-69 years old	6,320	(27.6)	18,961	(27.6)	
≥70 years old	8,711	(38.0)	26,013	(37.8)	
Residential location					<0.001
Rural	18,336	(80.0)	54,305	(78.9)	
Urban	4,595	(20.0)	14,488	(21.1)	
Social security type					<0.001
Medical aid	1,413	(6.2)	3,583	(5.2)	
Insurance (regional)	7,495	(32.7)	22,896	(33.3)	
Insurance (corporate)	14,023	(61.2)	42,314	(61.5)	
Income level					<0.001
Low	4,606	(20.1)	12,808	(18.6)	
Lower-middle	2,952	(12.9)	8,743	(12.7)	
Middle	3,581	(15.6)	10,687	(15.5)	
Upper-middle	4,620	(20.1)	14,287	(20.8)	
High	7,172	(31.3)	22,268	(32.4)	
CCI					<0.001
0	2,150	(9.4)	29,577	(43.0)	
1	2,759	(12.0)	19,290	(28.0)	
2	4,495	(19.6)	10,066	(14.6)	
≥3	13,527	(59.0)	9,860	(14.3)	
PPIs					0.016
Nonusers	15,397	(67.1)	45,473	(66.1)	
<30 days/year	7,053	(30.8)	21,774	(31.7)	
30-59 days/year	285	(1.2)	972	(1.4)	
60-89 days/year	111	(0.5)	298	(0.4)	
≥90 days/year	85	(0.4)	276	(0.4)	
NSAIDs					<0.001
Nonusers	877	(3.8)	3,056	(4.4)	
<30 days/year	13,955	(60.9)	40,559	(59.0)	
30-59 days/year	2,420	(10.6)	7,395	(10.8)	
60-89 days/year	1,262	(5.5)	3,821	(5.6)	
≥90 days/year	4,417	(19.3)	13,962	(20.3)	
Disability					0.021
No	19,995	(87.2)	60,386	(87.8)	
Yes	2,936	(12.8)	8,407	(12.2)	
Hypertension					<0.001
No	16,218	(70.7)	46,007	(66.9)	
Yes	6,713	(29.3)	22,786	(33.1)	
Diabetes mellitus					<0.001
No	18,562	(80.9)	59,052	(85.8)	
Yes	4,369	(19.1)	9,741	(14.2)	
Dyslipidemia					<0.001
No	20,641	(90.0)	60,278	(87.6)	
Yes	2,290	(10.0)	8,515	(12.4)	

CCI, Charlson Comorbidity Index; PPIs, proton pump inhibitors; NSAIDs, nonsteroidal anti-inflammatory drugs

a p-value from Chi-squared test.

### 3.2 Effect of ranitidine and nizatidine on GI cancer

Univariable conditional logistic regression analyses showed that ranitidine and/or nizatidine users had higher odds ratios (ORs) of GI cancer than nonusers in all average prescription day groups (< 30 days/year: OR [95% CI] = 1.04 [1.00-1.07]; 30–59 days/year: OR [95% CI] = 1.07 [0.98-1.17]; 60–89 days/year: OR [95% CI] = 1.22 [1.07-1.38]; ≥ 90 days/year: OR [95% CI] = 1.15 [1.02-1.30]). The ORs sharply increased as the CCI score increased. Subjects who took PPIs for < 30 days (OR [95% CI] = 0.95 [0.92–0.98]) and 30-59 days (OR [95% CI] = 0.86 [0.75–0.98]) had lower ORs of GI cancer than nonusers.

Multivariable analysis showed that ranitidine and/or nizatidine users had lower adjusted odds ratios (aORs) of GI cancer than nonusers in all average prescription day groups (< 30 days/year: aOR [95% CI] = 0.79 [0.75-0.82]; 30–59 days/year: aOR [95% CI] = 0.66 [0.59-0.73]; 60–89 days/year: aOR [95% CI] = 0.69 [0.59-0.81]; ≥ 90 days/year: aOR [95% CI] = 0.69 [0.59-0.79]). The aORs were higher in subjects with regional or corporate insurance than medical aid beneficiaries. However, subjects with higher incomes had lower aORs than those with a low income. The higher the CCI score, the higher the aOR for GI cancer. Subjects who had used PPIs had lower aORs for GI cancer than nonusers in all average prescription day groups (< 30 days/year: aOR [95% CI] = 0.75 [0.72-0.78]; 30–59 days/year: aOR [95% CI] = 0.54 [0.46-0.64]; 60–89 days/year: aOR [95% CI] = 0.79 [0.61-1.02]; ≥ 90 days/year: aOR [95% CI] = 0.65 [0.49-0.86]). The longer average prescription days per year of NSAIDs, the lower aORs were shown (< 30 days/year: aOR [95% CI] = 0.74 [0.66-0.82]; 30–59 days/year: aOR [95% CI] = 0.49 [0.43-0.55]; 60–89 days/year: aOR [95% CI] = 0.47 [0.41-0.54]; ≥ 90 days/year: aOR [95% CI] = 0.41 [0.36-0.46]). Subjects with disability, hypertension, DM, or dyslipidemia had lower aORs of GI cancer than those who did not have these conditions (**Table 2**).

**Table 2:** Conditional logistic regression analysis (*N* = 91,724).

Variables	Univariable			Multivariable		
	OR	(95% CI)	*p*	aOR	(95% CI)	*p*
Ranitidine and/or Nizatidine						
Nonusers	1.00			1.00		
<30 days/year	1.04	(1.00-1.07)	0.044	0.79	(0.75 - 0.82)	<0.001
30-59 days/year	1.07	(0.98-1.17)	0.112	0.66	(0.59-0.73)	<0.001
60-89 days/year	1.22	(1.07-1.38)	0.002	0.69	(0.59-0.81)	<0.001
≥90 days/year	1.15	(1.02-1.30)	0.019	0.69	(0.59-0.79)	<0.001
Residential location						
Rural	1.00			1.00		
Urban	0.94	(0.91-0.98)	<0.001	0.99	(0.95-1.04)	0.768
Social security type						
Medical aid	1.00			1.00		
Insurance (regional)	0.83	(0.77-0.88)	<0.001	1.09	(1.00-1.20)	0.051
Insurance (corporate)	0.84	(0.78-0.89)	<0.001	1.13	(1.03-1.23)	0.007
Income level						
Low	1.00			1.00		
Lower-middle	0.94	(0.89-0.99)	0.021	1.00	(0.93-1.08)	0.939
Middle	0.93	(0.89-0.98)	0.006	0.96	(0.90-1.03)	0.217
Upper-middle	0.90	(0.86-0.94)	<0.001	0.90	(0.84-0.95)	<0.001
High	0.89	(0.86-0.93)	<0.001	0.87	(0.82-0.92)	<0.001
CCI						
0	1.00			1.00		
1	2.17	(2.04-2.31)	<0.001	2.77	(2.60-2.96)	<0.001
2	6.72	(6.33-7.14)	<0.001	9.55	(8.95-10.19)	<0.001
≥3	21.86	(20.63-23.16)	<0.001	37.60	(35.21-40.16)	<0.001
PPIs						
Nonusers	1.00			1.00		
<30 days/year	0.95	(0.92-0.98)	0.003	0.75	(0.72-0.78)	<0.001
30-59 days/year	0.86	(0.75-0.98)	0.024	0.54	(0.46-0.64)	<0.001
60-89 days/year	1.09	(0.87-1.35)	0.465	0.79	(0.61-1.02)	0.072
≥90 days/year	0.90	(0.70-1.15)	0.392	0.65	(0.49-0.86)	0.003
NSAIDs						
Nonusers	1.00			1.00		
<30 days/year	1.21	(1.12-1.31)	<0.001	0.74	(0.66-0.82)	<0.001
30-59 days/year	1.14	(1.04-1.25)	0.005	0.49	(0.43-0.55)	<0.001
60-89 days/year	1.15	(1.04-1.27)	0.009	0.47	(0.41-0.54)	<0.001
≥90 days/year	1.10	(1.01-1.20)	0.039	0.41	(0.36-0.46)	<0.001
Disability						
No	1.00			1.00		
Yes	1.06	(1.01-1.11)	0.018	0.86	(0.81-0.91)	<0.001
Hypertension						
No	1.00			1.00		
Yes	0.82	(0.79-0.85)	<0.001	0.74	(0.71-0.77)	<0.001
Diabetes mellitus						
No	1.00			1.00		
Yes	1.45	(1.39-1.51)	<0.001	0.60	(0.57-0.63)	<0.001
Dyslipidemia						
No	1.00			1.00		
Yes	0.78	(0.74-0.82)	<0.001	0.65	(0.61-0.69)	<0.001

OR, odds ratio; aOR, adjusted odds ratio; CI, confidence interval; CCI, Charlson Comorbidity Index; PPIs, proton pump inhibitors; NSAIDs, nonsteroidal anti-inflammatory drugs.

### 3.3 Sensitivity analyses

To validate the lag periods, sensitivity tests were performed. As the lag periods became longer, there were fewer drug users. The proportion of ranitidine and/or nizatidine users was 58.1% in the case group and 58.0% in the control groups with a 365-day lag period (*p =* 0.027). In total, 52.1% of the case group were drug users, compared with 52.9% of the controls, with a 730-day lag period (*p =* 0.011) (**Supplementary Table 1**). The results were the same with a lag period of 180 days according to multivariable conditional logistic regression analyses. Compared to the nonusers, ranitidine and/or nizatidine users showed lower aORs in all average prescription day groups, in both the 365-day (< 30 days/year: aOR [95% CI] = 0.77 [0.74-0.81]; 30–59 days/year: aOR [95% CI] = 0.64 [0.57-0.72]; 60–89 days/year: aOR [95% CI] = 0.70 [0.59-0.82]; ≥ 90 days/year: aOR [95% CI] = 0.70 [0.60-0.81]) and 730-day (< 30 days/year: aOR [95% CI] = 0.77 [0.73-0.80]; 30–59 days/year: aOR [95% CI] = 0.66 [0.59-0.74]; 60–89 days/year: aOR [95% CI] = 0.69 [0.58-0.81]; ≥ 90 days/year: aOR [95% CI] = 0.70 [0.60-0.81]) lag periods (**Supplementary Table 2**).

### 3.4 Additional analyses

**Figure 3:**
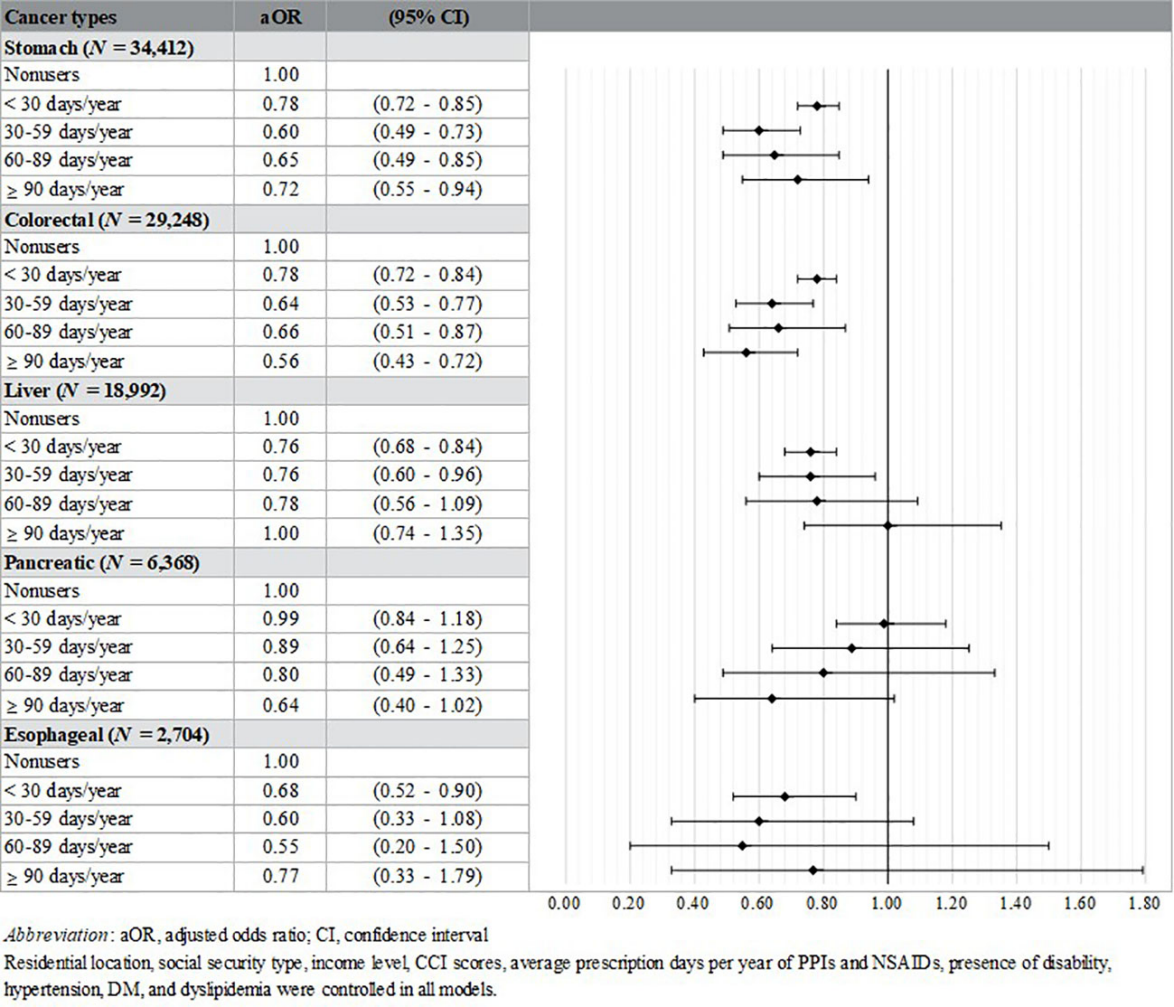
Additional analysis by cancer types.

Additional analyses were performed by cases’ cancer type (stomach, *N =* 34,412; colorectal, *N =* 29,248; liver, *N =* 18,992; pancreatic, *N =* 6,368; esophageal, *N =* 2,704). Controls were divided according to the cancer types of matched cases. The aORs of the stomach (< 30 days/year: aOR [95% CI] = 0.78 [0.72-0.85]; 30–59 days/year: aOR [95% CI] = 0.60 [0.49-0.73]; 60–89 days/year: aOR [95% CI] = 0.65 [0.49-0.85]; ≥ 90 days/year: aOR [95% CI] = 0.72 [0.55-0.94]) and colorectal cancer groups (< 30 days/year: aOR [95% CI] = 0.78 [0.72-0.84]; 30–59 days/year: aOR [95% CI] = 0.64 [0.53-0.77]; 60–89 days/year: aOR [95% CI] = 0.66 [0.51-0.87]; ≥ 90 days/year: aOR [95% CI] = 0.56 [0.43-0.72]) showed significantly lower results in all prescription days than nonusers. And the subjects who took the drug less than 30 days (aOR [95% CI] = 0.76 [0.68-0.84]) and 30-59 days (aOR [95% CI] = 0.76 [0.60-0.96]) in the liver cancer group had lower aOR than nonusers. Lastly, only < 30 days significant results in the esophageal cancer group (aOR [95% CI] = 0.68 [0.52-0.90]) (**Figure 3**).

Also, we had unconditional logistic analyses stratified by the CCI to evaluate the presence of effect modification (CCI = 0, *N* = 31,727; CCI = 1, *N* = 22,049; CCI = 2, *N* = 14,561; CCI ≥ 3, *N* = 23,387). Within the subgroup of CCI = 0, the analysis revealed that individuals who took the drugs for ≥ 90 days (aOR [95% CI] = 1.86 [1.08-3.20]) exhibited higher aOR compared to nonusers, with age and sex controlled as covariates. However, the subgroups with higher CCI scores exhibited lower aORs among drug users compared to nonusers. Furthermore, after controlling for covariates including sociodemographic status and comorbidities, drug users consistently displayed significantly lower aORs than nonusers across all subgroups based on CCI score (**Supplementary Table 3**).

## 4 Discussion

A few years ago, the FDA announced that ranitidine and nizatidine contained the carcinogen NDMA. A follow-up study was then conducted using FDA Adverse Event Reporting System (FAERS) data from the United States, and showed that the proportion of adverse effects, especially GI cancer, was significantly higher in ranitidine users than in users of comparable drugs (PPIs and other H2RAs) (22). However, the same results were not obtained in subsequent studies. A randomized, double-blind, placebo-controlled, crossover clinical trial study that analyzed the NDMA concentration in urine 24 hours after taking the drug did not find a statistically significant difference between ranitidine and placebo groups (23). An *in vitro* study that observed the formation of NDMA after adding ranitidine tablets to simulated gastric fluid confirmed the formation of the carcinogen through liquid chromatography–mass spectrometry (LC-MS). However, as the solution was highly acidic compared to actual gastric acid, the results may not be applicable to the general population (24). Furthermore, a Danish study using prescription registry data and conducting survival analysis, concluded that there was no evidence of carcinogenic effects on esophageal cancer, stomach cancer, liver cancer, and pancreatic cancer in individuals who had taken ranitidine (25).

In this study, the incidence of GI cancer in the case group taking ranitidine and nizatidine was lower than in nonusers, regardless of the average prescription days per year. Also, patients taking PPIs had lower aORs of GI cancer compared to nonusers. In additional analyses conducted according to cases’ cancer type, drug users typically showed lower aORs of cancer incidence than nonusers. We believe that the differences in results among studies can be explained as follows.

First, the study settings differed. As cancer (the main outcome of this study) has a longer latent period than other diseases (26–28), it is important to maximize the follow-up period after exposure to a specific drug. Moreover, sociodemographic factors and health behavior may modify relationships between drugs and diseases, so must to be considered (29). But, the studies reporting an association between ranitidine and cancer development used self-report or cross-sectional data, and did not consider the other health conditions of their subjects (22, 30). In this study, the ORs of GI cancer of ranitidine and/or nizatidine users were higher than those of nonusers in univariable analysis. However, when subjects’ socioeconomic status, CCI, PPIs and NSAIDs uses, presence of disability and comorbidities (hypertension, DM, and dyslipidemia) were considered, the opposite result was obtained. Thus, we conducted an analysis to investigate the factors influencing the change in associations, employing a stepwise approach in which variables were incrementally added to the model. Our aim was to determine which covariates influenced the change in direction. Notably, we observed a shift in the association direction when considering the CCI score. Consequently, we proceeded to perform subgroup analyses based on this score. Initially, when controlling for age and sex only, we identified a higher aOR within the CCI = 0 group in relation to subjects who had taken the drugs for more than 90 days. However, upon accounting for all the covariates employed in this study, the previously significant risky direction became non-significant, indicating a change in the observed relationship.

The change from a positive association to a protective association could be due to the confounding effect of one or more of the covariates that were controlled for in the analysis (31). In other words, the covariates may have influenced the observed association between the drugs and GI cancer, resulting in a more positive association in the univariable model but, less so in the multivariable model. Based on the covariates included in the model, it is possible that some of them have a confounding effect on the association between the drugs and GI cancer. For example, socioeconomic statuses, such as social security type and income level, may affect the probability of receiving a diagnosis of GI cancer or accessing healthcare (32–38), which in turn could influence the likelihood of being prescribed ranitidine or nizatidine. Similarly, the presence of comorbidities such as hypertension, diabetes mellitus, and dyslipidemia may be associated with both the intake of the drugs and the risk of GI cancer (39–44).

A Swedish study using four nationwide Swedish registries (the Swedish Prescribed Drug Registry, the Swedish Cancer Registry, the Swedish Patient Registry, and the Swedish Causes of Death Registry) drew conclusions similar to our study. They assessed whether H2RAs and PPIs influenced gastric cancer incidence, and found that the risk was not increased by long-term use of acid suppressants. The authors suggested that long-term use of acid suppressants may be beneficial, by improving risk factors for gastric cancer such as peptic ulcers and *Helicobacter pylori* (*H. pylori*) (45). Furthermore, we divided the subject into five groups according to their cancer types, because NDMA exposure is known to be more harmful to the liver than other organs (46). As the results of the additional analyses, most of the cancer types showed lower risk in the drug users than nonusers. This was the same pattern as the result of our main analysis.

The characteristics of the subjects were also differed among studies. The study described above analyzed adverse events only in patients who had taken ranitidine, and thus had already experienced digestive diseases (45). According to previous studies conducted clinical review or meta-analysis, people who have experienced digestive diseases such as gastritis, peptic ulcer, pancreatitis, and GERD have a higher risk of GI cancer (47–49). Therefore, selection bias may have occurred (50). This also applies to studies limited in terms of the age or sex of their subjects. However, in our study, data from 1 million people randomly selected from the Korean population were used, with no restrictions imposed in terms of the other conditions; this represents a strength of our study.

Another strength of this study was that we matched the cases and controls by their sex and age. Sex is a very important factor in pharmacokinetics and pharmacodynamics (51, 52). Also, age is highly influential in terms of the incidence of cancer (53). Through this, we tried to reduce the heterogeneity of subjects and to control the confounding effects of sex and age by extracting case-control groups with similar characteristics (54).

We used a different analysis method than previous studies, to control the selection and immortal time biases, a nested case-control study design was applied. Lag periods were also considered to reduce the likelihood of reverse causation (29). However, there are several limitations that should be taken into consideration. First, the data used in this study were health insurance claims data; if a patient purchases ranitidine or nizatidine from a pharmacy without a doctor’s prescription, the data may not be recorded. Thus, only the drugs that were available as prescriptions were analyzed in this study. Further research is needed covering the OTC use of the drugs. Second, the data from clinical and health examinations were not used. Smoking, drinking, eating habits, and BMI are closely related to GI cancer (1, 55, 56). These factors were not considered because our cohort included a very small number of participants with health examination data. To overcome this, efforts were made to reduce the influence of variables that could not be considered when calculating the CCI, and by determining whether hypertension, DM, and dyslipidemia were present.

## 5 Conclusions

Our study suggests that while there is no evidence that ranitidine and nizatidine increase the risk of GI cancer, the use of these acid suppressants may reduce the risk of GI cancer by improving digestive diseases. However, this finding should be interpreted with caution as other factors such as socioeconomic status, health behaviors, and comorbidities may influence the association between the drugs and GI cancer risk.

Our study provides valuable information for clinicians in making informed decisions about the use of these medications in treating digestive disorders. Further research is warranted to investigate the potential positive association between these drugs and GI cancer.

## Data availability statement

The raw data supporting the conclusions of this article will be made available by the authors, without undue reservation.

## Ethics statement

This study was approved by the Institutional Review Board (IRB) of the Yonsei University Health System, Severance Hospital (IRB No. 4-2021-0727). Patient consent was waived because anonymized data was provided by NHIS.

## Author contributions

Conceptualization, HK and SP; methodology, HK, SP, CN, and D-WC; validation, SP, CN, and D-WC; formal analysis, HK; investigation, HK; writing—original draft preparation, HK; writing—review and editing, SP, CN, and D-WC; visualization, HK; supervision, SP; project administration, SP; funding acquisition, SP. All authors contributed to the article and approved the submitted version.
