# Supplementary material for: Effects of ranitidine and nizatidine on the risk of gastrointestinal cancer
Source: Front Oncol. 2023 Jul 27;13:1182174. doi: 10.3389/fonc.2023.1182174 (PMC10415903; doi:10.3389/fonc.2023.1182174)
Supplement: Supplementary file 1 [file Table_1.docx]

Supplementary Material

Effects of Ranitidine and Nizatidine on the Risk of Gastrointestinal Cancer

**Hyejung Kang^1^, Chung Mo Nam^2^, Dong-Woo Choi^3^ and Sohee Park^1^***

*** Correspondence: Sohee Park: soheepark@yuhs.ac**

# Supplementary Tables

**Supplementary Table 1.** Distribution of the subjects according to lag periods.

| **Variables, *N* (%)** | **Lag periods** | | | | | | | | | | |
| --- | --- | --- | --- | --- | --- | --- | --- | --- | --- | --- | --- |
|  | **365 days** | | | | | **730 days** | | | | | |
|  | **Cases** | | **Controls** | | ***p* ^a^** | **Cases** | | **Controls** | | ***p* ^a^** | |
|  | **(*N* = 22,931)** | | **(*N* = 68,793)** | |  | **(*N* = 22,931)** | | **(*N* = 68,793)** | |  |  |
| **Ranitidine and/or Nizatidine** |  |  |  |  | 0.027 |  |  |  |  | 0.011 | |
| Nonusers | 9,613 | (41.9) | 28,857 | (42.0) |  | 10,983 | (47.9) | 32,404 | (47.1) |  | |
| < 30 days/year | 11,898 | (51.9) | 35,946 | (52.3) |  | 10,686 | (46.6) | 32,857 | (47.8) |  | |
| 30-59 days/year | 712 | (3.1) | 2,148 | (3.1) |  | 641 | (2.8) | 1,864 | (2.7) |  | |
| 60-89 days/year | 332 | (1.4) | 851 | (1.2) |  | 288 | (1.3) | 772 | (1.1) |  | |
| ≥ 90 days/year | 376 | (1.6) | 991 | (1.4) |  | 333 | (1.5) | 896 | (1.3) |  | |
| **PPIs** |  |  |  |  | <0.001 |  |  |  |  | <0.001 | |
| Nonusers | 15,937 | (69.5) | 46,816 | (68.1) |  | 16,857 | (73.5) | 49,328 | (71.7) |  | |
| < 30 days/year | 6,565 | (28.6) | 20,562 | (29.9) |  | 5,727 | (25.0) | 18,267 | (26.6) |  | |
| 30-59 days/year | 252 | (1.1) | 902 | (1.3) |  | 205 | (0.9) | 778 | (1.1) |  | |
| 60-89 days/year | 104 | (0.5) | 268 | (0.4) |  | 81 | (0.4) | 219 | (0.3) |  | |
| ≥ 90 days/year | 73 | (0.3) | 245 | (0.4) |  | 61 | (0.3) | 201 | (0.3) |  | |
| **NSAIDs** |  |  |  |  | <0.001 |  |  |  |  | <0.001 | |
| Nonusers | 1,048 | (4.6) | 3,551 | (5.2) |  | 1,636 | (7.1) | 5,153 | (7.5) |  | |
| < 30 days/year | 14,092 | (61.5) | 40,847 | (59.4) |  | 14,064 | (61.3) | 40,918 | (59.5) |  | |
| 30-59 days/year | 2,320 | (10.1) | 7,339 | (10.7) |  | 2,204 | (9.6) | 7,034 | (10.2) |  | |
| 60-89 days/year | 1,221 | (5.3) | 3,641 | (5.3) |  | 1,108 | (4.8) | 3,293 | (4.8) |  | |
| ≥ 90 days/year | 4,250 | (18.5) | 13,415 | (19.5) |  | 3,919 | (17.1) | 12,395 | (18.0) |  | |
| *Abbreviation*: PPIs, proton pump inhibitors; NSAIDs, nonsteroidal anti-inflammatory drugs | | | | | | | | | | |  |
| ^a^ *p*-value from Chi-squared test | | | | | | | | | | |  |

**Supplementary Table 2.** Conditional logistic regression analysis according to lag periods.

| **Variables** | **Lag periods** | | | | | | | | | |  |
| --- | --- | --- | --- | --- | --- | --- | --- | --- | --- | --- | --- |
|  | **365 days** | | | | | **730 days** | | | | |  |
|  | **(N = 91,724)** | | | | | **(N = 91,724)** | | | | |  |
|  | **aOR** | **(95% CI)** | | | ***p*** | **aOR** | **(95% CI)** | | | ***p*** |  |
| **Ranitidine and/or Nizatidine** |  |  |  |  |  |  |  |  |  |  |  |
| Nonusers | 1.00 |  |  |  |  | 1.00 |  |  |  |  |  |
| < 30 days/year | 0.77 | (0.74 | - | 0.81) | <0.001 | 0.77 | (0.73 | - | 0.80) | <0.001 |  |
| 30-59 days/year | 0.64 | (0.57 | - | 0.72) | <0.001 | 0.66 | (0.59 | - | 0.74) | <0.001 |  |
| 60-89 days/year | 0.70 | (0.59 | - | 0.82) | <0.001 | 0.69 | (0.58 | - | 0.81) | <0.001 |  |
| ≥ 90 days/year | 0.70 | (0.60 | - | 0.81) | <0.001 | 0.70 | (0.60 | - | 0.81) | <0.001 |  |
| **PPIs** |  |  |  |  |  |  |  |  |  |  |  |
| Nonusers | 1.00 |  |  |  |  | 1.00 |  |  |  |  |  |
| < 30 days/year | 0.76 | (0.72 | - | 0.79) | <0.001 | 0.77 | (0.73 | - | 0.80) | <0.001 |  |
| 30-59 days/year | 0.52 | (0.44 | - | 0.61) | <0.001 | 0.50 | (0.41 | - | 0.60) | <0.001 |  |
| 60-89 days/year | 0.85 | (0.65 | - | 1.11) | 0.241 | 0.84 | (0.62 | - | 1.12) | 0.238 |  |
| ≥ 90 days/year | 0.68 | (0.50 | - | 0.92) | 0.012 | 0.72 | (0.52 | - | 1.01) | 0.055 |  |
| **NSAIDs** |  |  |  |  |  |  |  |  |  |  |  |
| Nonusers | 1.00 |  |  |  |  | 1.00 |  |  |  |  |  |
| < 30 days/year | 0.75 | (0.68 | - | 0.83) | <0.001 | 0.77 | (0.71 | - | 0.84) | <0.001 |  |
| 30-59 days/year | 0.50 | (0.44 | - | 0.56) | <0.001 | 0.52 | (0.47 | - | 0.58) | <0.001 |  |
| 60-89 days/year | 0.48 | (0.43 | - | 0.55) | <0.001 | 0.52 | (0.46 | - | 0.59) | <0.001 |  |
| ≥ 90 days/year | 0.43 | (0.38 | - | 0.48) | <0.001 | 0.46 | (0.42 | - | 0.51) | <0.001 |  |
| *Abbreviation*: aOR, adjusted odds ratio; CI, confidence interval; PPIs, proton pump inhibitors; NSAIDs, nonsteroidal anti-inflammatory drugs | | | | | | | | | | | |
| Residential location, social security type, income level, presence of disability, hypertension, diabetes mellitus, and dyslipidemia were controlled. | | | | | | | | | | | |

**Supplementary Table 3.** Subgroup analysis by CCI scores (unconditional logistic regression model).

| **Ranitidine and/or Nizatidine** | **Age and Sex were controlled** | | | | | **Multivariable*** | | | | |  |
| --- | --- | --- | --- | --- | --- | --- | --- | --- | --- | --- | --- |
|  | **aOR** | **(95% CI)** | | | ***p*** | **aOR** | **(95% CI)** | | | ***p*** |  |
| **CCI = 0 (*N* = 31,727)** |  |  |  |  |  |  |  |  |  |  |  |
| Nonusers | 1.00 |  |  |  |  | 1.00 |  |  |  |  |  |
| < 30 days/year | 0.96 | (0.88 | - | 1.05) | 0.396 | 1.01 | (0.91 | - | 1.12) | 0.858 |  |
| 30-59 days/year | 1.13 | (0.77 | - | 1.65) | 0.547 | 1.01 | (0.67 | - | 1.50) | 0.977 |  |
| 60-89 days/year | 1.67 | (0.91 | - | 3.05) | 0.096 | 1.45 | (0.78 | - | 2.71) | 0.239 |  |
| ≥ 90 days/year | 1.86 | (1.08 | - | 3.20) | 0.025 | 1.62 | (0.92 | - | 2.83) | 0.239 |  |
| **CCI = 1 (*N* = 22,049)** |  |  |  |  |  |  |  |  |  |  |  |
| Nonusers | 1.00 |  |  |  |  | 1.00 |  |  |  |  |  |
| < 30 days/year | 0.76 | (0.70 | - | 0.83) | <0.001 | 0.89 | (0.82 | - | 0.98) | 0.013 |  |
| 30-59 days/year | 0.74 | (0.58 | - | 0.93) | 0.012 | 0.93 | (0.73 | - | 1.19) | 0.580 |  |
| 60-89 days/year | 0.91 | (0.64 | - | 1.31) | 0.621 | 1.19 | (0.82 | - | 1.73) | 0.357 |  |
| ≥ 90 days/year | 0.80 | (0.56 | - | 1.14) | 0.211 | 1.04 | (0.72 | - | 1.49) | 0.837 |  |
| **CCI = 2 (*N* = 14,561)** |  |  |  |  |  |  |  |  |  |  |  |
| Nonusers | 1.00 |  |  |  |  | 1.00 |  |  |  |  |  |
| < 30 days/year | 0.51 | (0.47 | - | 0.54) | <0.001 | 0.70 | (0.64 | - | 0.76) | <0.001 |  |
| 30-59 days/year | 0.33 | (0.27 | - | 0.40) | <0.001 | 0.66 | (0.53 | - | 0.82) | <0.001 |  |
| 60-89 days/year | 0.29 | (0.21 | - | 0.40) | <0.001 | 0.63 | (0.45 | - | 0.89) | 0.007 |  |
| ≥ 90 days/year | 0.20 | (0.15 | - | 0.28) | <0.001 | 0.43 | (0.31 | - | 0.61) | <0.001 |  |
| **CCI ≥** **3 (*N* = 23,387)** |  |  |  |  |  |  |  |  |  |  |  |
| Nonusers | 1.00 |  |  |  |  | 1.00 |  |  |  |  |  |
| < 30 days/year | 0.63 | (0.60 | - | 0.67) | <0.001 | 0.86 | (0.80 | - | 0.92) | <0.001 |  |
| 30-59 days/year | 0.37 | (0.33 | - | 0.42) | <0.001 | 0.69 | (0.60 | - | 0.79) | <0.001 |  |
| 60-89 days/year | 0.36 | (0.31 | - | 0.43) | <0.001 | 0.73 | (0.61 | - | 0.87) | <0.001 |  |
| ≥ 90 days/year | 0.37 | (0.32 | - | 0.43) | <0.001 | 0.75 | (0.63 | - | 0.89) | <0.001 |  |
| *Abbreviation*: aOR, adjusted odds ratio; CI, confidence interval; CCI, Charlson Comorbidity Index | | | | | | | | | | | |
| * Age, sex, residential location, social security type, income level, average prescription days per year of PPIs, NSAIDs, presence of disability, hypertension, diabetes mellitus, and dyslipidemia were controlled in all models. | | | | | | | | | | | |
